# Sciatic obturator femoral technique versus spinal anaesthesia in patients undergoing surgery for fixation of open tibial fractures using Ilizarov external fixator. A randomised trial

**DOI:** 10.1186/s12871-019-0920-6

**Published:** 2020-01-04

**Authors:** Hoda Shokri, Amr A. Kasem

**Affiliations:** 1 Assistant Professor of Anaesthesiology, Ain Shams University, Cairo, Egypt; 2 Lecturer of Anaesthesiology, Ain Shams University, Cairo, Egypt

**Keywords:** Spinal anesthesia, SOFT block, Ilizarov, Analgesia

## Abstract

**Background:**

Peripheral nerve block is preferable for lower extremity surgery because it sufficiently blocks pain pathways at different levels providing excellent anaesthesia at the site of surgery. We designed this study to compare the efficacy and safety of SOFT block (sciatic-obturator-femoral technique) compared with spinal anaesthesia in patients undergoing surgery for fixation of open tibial fractures using Ilizarov external fixator.

**Methods:**

One hundred and seven patients ASA I, II scheduled for fixation of open tibial fractures using Ilizarov external fixator. The patients were randomly allocated to receive either spinal anaesthesia or SOFT block. In spinal anaesthesia group, patients received spinal anaesthesia with hyperbaric bupivacaine 0.5% (7. 5-10mg). In SOFT group, patients received SOFT block with bupivacaine 0.25%. Primary endpoint included the duration of analgesia. The secondary endpoints included patient satisfaction scores, visual analogue scores, incidence of adverse events as vomiting, systemic toxicity from local anaesthetic drug and time to first effect of the techniques.

**Results:**

The duration of SOFT block and time to first analgesic dose in SOFT group was significantly longer (*p* < 0.001). There was no significant difference between the study groups regarding satisfaction scores, the incidence of cardiovascular collapse, seizures and paraesthesia. Pain scores were significantly lower in SOFT group at 3,6,12 h postoperative (*p* < 0.001). The time to the first effect was significantly longer in SOFT group (p < 0.001).

**Conclusion:**

SOFT is a feasible technique of local anaesthesia for control of postoperative pain with unremarkable adverse events compared with spinal anaesthesia, in patients undergoing fixation of tibial fractures using Ilizarov external fixator.

**Trial registration:**

This trial was retrospectively registered at ClinicalTrials.gov. registry number: NCT03450798 on February 20, 2018.

## Background

Peripheral nerve block is a popular pain control tool for the lower limb procedures because it lowers the pain scores, reduces opioid requirements and improves patient’s satisfaction [1]. On the contrary to different anesthetic techniques, as spinal or general anesthesia, properly performed peripheral nerve blocks are associated with uneventful recovery, brilliantly treat post-operative pain inflicting early hospital discharge [2]. Extra blessings of peripheral nerve blocks are that they can be used in patients receiving anti-coagulants or tormented by lumbo-sacral unwellness and also neuroaxial anaesthesia can be done without airway instrumentation as well [3].

Recently, there is a big interest in regional anesthesia particularly peripheral nerve blockade [3] Research in this field has advanced a lot thanks to the availability of brilliant equipments used in different procedures. Femoral nerve block alone or combined with another peripheral nerve block is used for total knee replacement surgical procedures and has successfully treated postoperative pain bypassing any adverse events as cardiovascular or respiratory problems [4].

SOFT is a recent single-puncture block, it has shown better quality than the earlier approaches of blocking the sciatic, femoral, and obturator nerves and it takes less than 20 mins to perform [5].

We designed this prospective study to compare the efficacy and safety of SOFT compared with spinal anaesthesia in patients undergoing surgery for fixation of open tibial fractures using Ilizarov external fixator.

## Methods

After the approval of ethics committee of Ain Shams University, number FMASU R 10 / 2018, this study was registered in ClinicalTrials.gov: NCT03450798 and carried out according to the Consolidated Standards of Reporting Trials (CONSORT) 2010 statement [6].This prospective randomized double blinded parallel group study was conducted over 107 consecutive patients aged from 3 5- 57 years old, American Association of Anesthesiologists(ASA) I and II, scheduled for elective surgery for fixation of open tibial fractures using Ilizarov external fixator. This study was carried out at Ain Shams University hospitals from February 2018 to January 2019. Initially written informed consent was signed by all patients.

Patients unable to communicate with the investigators or hospital staff, morbidly obese patients (body mass index> 40 kg/m^2^), patients undergoing bilateral surgery, patients with coagulopathies, renal insufficiency (creatinine> 1.5 mg/dl), ASA III-IV, urgent procedures, contraindications to regional anesthesia, patients with unstable vital signs and patients with head or chest trauma were excluded from the study.

Pre-anaesthetic check, full history and routine investigations were done before the surgery.

After an intravenous (IV) cannula was secured, and midazolam 0.05 mg/kg IV was given to all patients before transfer to the operating room. Standard monitoring devices as electrocardiography, non- invasive blood pressure and pulse oximetry were placed. Then, patients were randomly allocated by sealed envelope technique done according to the randomisation schedule, prepared and opened by a resident not involved in any part of the study to receive either spinal anesthesia or SOFT block, the clinician in charge of data collection and patients’ follow-up was blinded to the patients’ grouping. A single experienced operator had performed all blocks and spinal anaesthesia and assessed their success did not participate in the study and was blinded to its nature. In spinal group, patients received spinal anaesthesia with hyperbaric bupivacaine (AstraZeneca, UK) 0.5% (7.5-10 mg) which was administered via a 25-G spinal needle at L4-L5 or L3-L4 while the patient positioned in the sitting position under complete aseptic conditions.

**Fig. 1:**
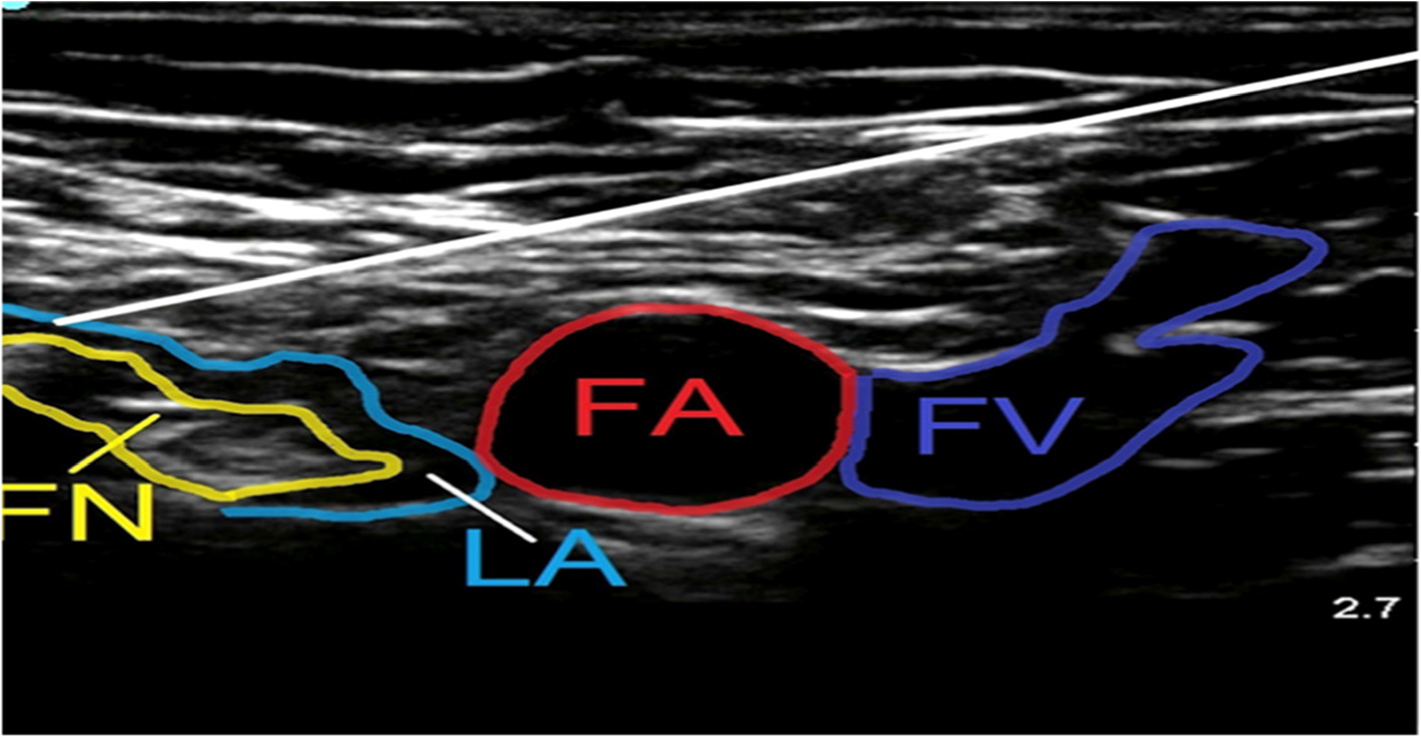
Ultrasound images obtained during femoral nerve block. Ultrasound image of the needle path to block the femoral nerve. The needle (white line) as shown pierces the fascia iliaca lateral to the femoral nerve (FN) marked by yellow arrow and the needle tip is advanced along the deep border of the nerve. FA, femoral artery

**Fig. 2:**
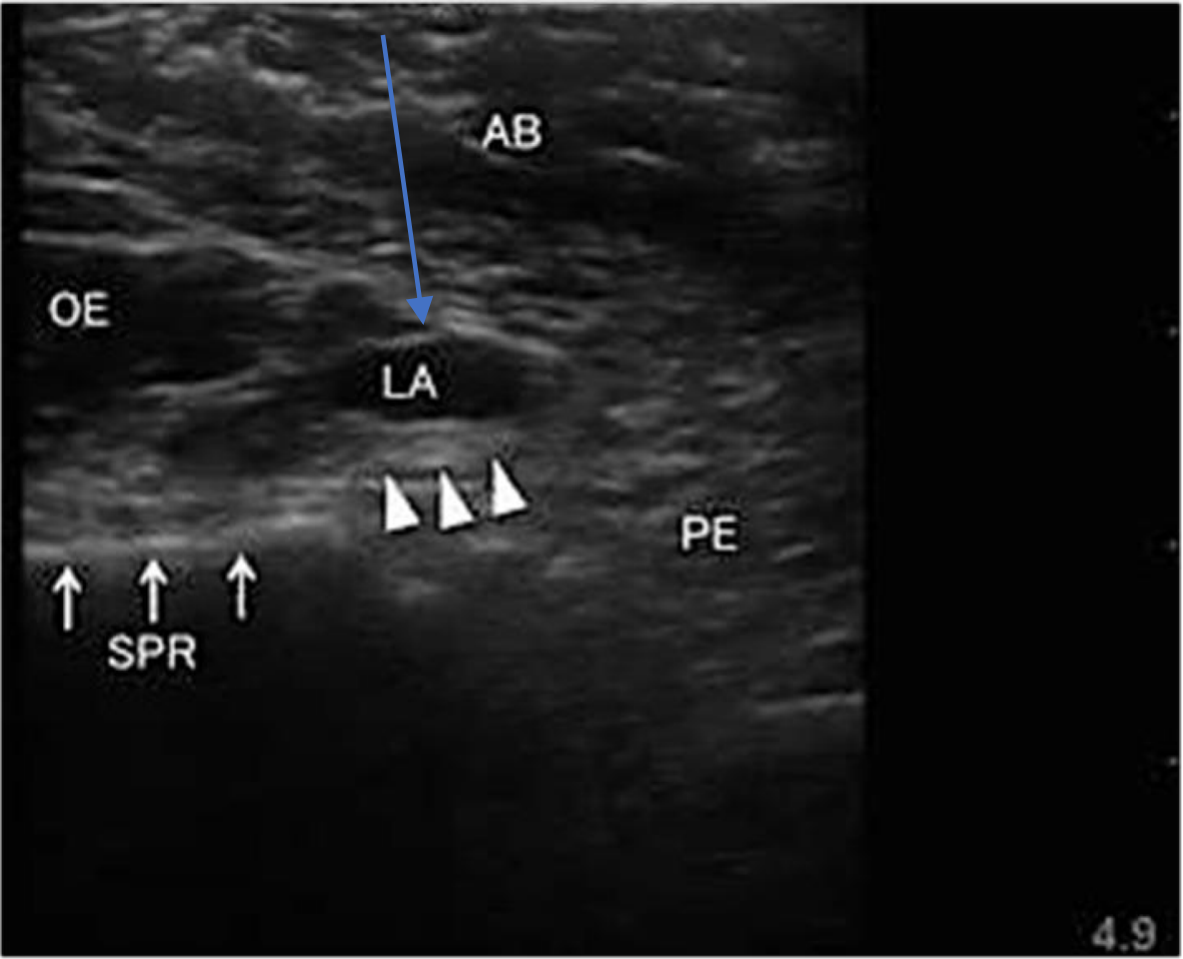
Ultrasound images obtained during obturator nerve block. The probe was shifted medially and directed cranially. A hyperechoic thick fascia between the pectineus and obturator externus muscles (open triangles) is the target plane. The needle (blue arrow) was moved toward the fascial plane deep to the pectineus (PE) muscle using an out of plane method. LA: local anaesthetic; AB: anterior branch of obturator nerve; SPR: superior pubic ramus (arrows); OE: obturator externus

**Fig. 3:**
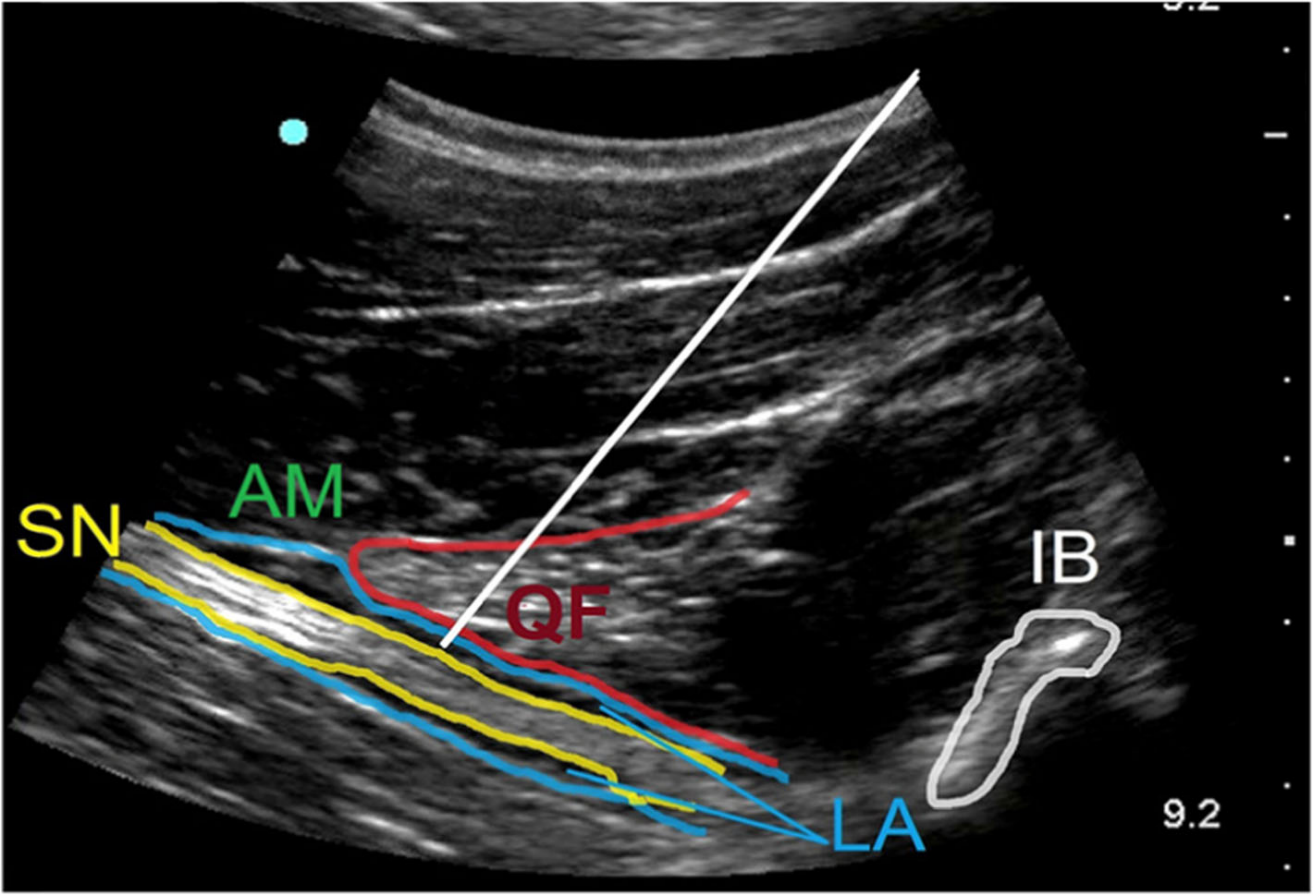
Ultrasound images obtained during sciatic nerve block. The curved probe is put vertically, inferior to the needle, to see the sciatic nerve (SN) clearly. The needle (white line) was directed by an in-plane technique toward the sciatic nerve deep to the inferior border of the quadratus femoris muscle to block the sciatic nerve deep to the quadratus femoris (QF) muscle. AM = adductor magnus; IB = ischium bone; LA = localanaesthetic injected; P = pectineus; QF = quadratus femoris

In SOFT group, patients received SOFT block where patients were positioned in supine position under complete aseptic conditions, a linear US probe (GE LOGIQe, Wauwatosa, Wisconsin, USA) was placed on the inguinal crease to clearly show the femoral nerve and vessels as shown in Fig. 1. After subcutaneous local anaesthetic wheal using bupivacaine 0.5% was made, a 12-cm stimulating block needle was introduced using an in-plane technique medial to the femoral vein and advanced 1-3 cm below and parallel to the skin. Then, it was redirected toward the fibres of the femoral nerve, where 15 mL of bupivacaine 0.25% was injected. To block the obturator nerve, the probe was shifted medially, superior to the needle and directed cranially to identify the pectineus muscle. The needle was then withdrawn to the subcutaneous tissue and redirected using an out-of-plane technique toward the deep surface of the pectineus without muscle twitches then 10 mL of bupivacaine (AstraZeneca, UK) 0.25% was injected slowly to ensure better spread of the local anaesthetic (LA) as shown in Fig. 2 [4]. To locate the sciatic nerve, the curvilinear probe was handled medial to the femoral vessels, inferior to the needle, and tilted to get the clearest image of the sciatic nerve. The needle was inserted then withdrawn subcutaneously and directed by an in-plane technique toward the sciatic nerve deep to the inferior border of the quadratus femoris muscle as shown in Fig. 3. Then 20 mL of bupivacaine 0.25% was injected after needle had elicited tibial twitches using 1 mA current. The techniques were evaluated every 5 min after completion of the technique for 20 min till successful block was achieved then surgical incision was allowed. Midazolam 2 mg doses (Dormicum 5 mg/ml; Roche Basel, Swizerland), 50 μg fentanyl (fentanyl 50 μg/ml, 2 ml; (ADVANZ Pharma, UK) and 15 mg ketamine (Ketalar, 50 mg/ml, 10 ml; Pfizer, Sandwich, UK) were given when sedo-analgesia was required. Successful SOFT was confirmed by patient’s inability to extend a fully flexed knee, to flex the foot and to adduct an abducted hip respectively. Baseline heart rate and blood pressure values were monitored every 5 min after block performance till the end of surgery. Hypotension was defined as a decrease in systolic arterial blood pressure by 20% or more from baseline values, and it was initially treated with 200 ml IV infusion of Ringer’s lactate solution; if this proved to be ineffective, an IV bolus of phenylephrine (40–50 mcg) was given. Bradycardia was defined as heart rate drops by more than 20% from the baseline values, and it was treated with 0.5 mg IV atropine.

At the end of the procedure, patients were transferred to the post-anaesthesia care unit where they were followed up 24 h after surgery by an experienced nurse and an end point assessor of the outcomes who were blinded to the nature of study and not part of it. Patient’s satisfaction was assessed using 4-point Likert scale through which we can assess the effectiveness of the block or spinal anaesthesia [7] (1 = very dissatisfied, 2 = unsatisfied, 3 = satisfied, 4= very satisfied) and potential complications. Visual analogue score scale was used for assessment of postoperative pain. It is a numerical rating scale where patients were taught to use this score to report the degree of postoperative pain from ‘0’ to ‘10’ with ‘0’ = no pain and ‘10’ = the worst imaginable pain. The same postoperative pain management protocol was followed for all patients. If pain score ≥ 4, patients were given fentanyl 50 μg i.v. bolus as a primary rescue analgesic which can be repeated after 1 h until pain score < 4. In addition to intravenous infusion of acetaminophen 15 mg/kg 6 hourly was administered as a secondary rescue analgesic.

### Primary endpoint

Included duration of analgesia: the time to the first analgesic requirement.

### Secondary endpoints

Included patient satisfaction scores, visual analogue scores, the incidence of adverse events as episodes of vomiting which was treated by metoclopramide 10 mg i.v. bolus, time to first effect of the technique, systemic toxicity of local anesthetics as seizures, cardiovascular collapse and persistent paraesthesia observed within 48 h of the block.

In case of failure of the block (persistence of pain sensation that requires completion of the procedure using either spinal or general anaesthesia) or incidence of seizures resulting from local anesthetic toxicity, general anaesthesia was given so those patients were excluded from the study.

In case of, cardiovascular collapse, the patients were treated with intravenous fluids and vasopressors.

### Sample size calculation

Using Power Calculations and Sample Size software (PASS; NCSS, LLC, East Kaysville, UT, USA) revealed that 120 patients, 60 per arm, were needed after considering a 10% drop out (power of 80%; alpha error at 5%). These calculations were based on a previous study [8] that showed that.

the time to first rescue analgesic requirement (duration of analgesia) for combined sciatic and femoral block group was 336 ± 18 min.

### Statistical analysis

The data were analysed using IBM SPSS Statistics for Windows (Version 23.0. Armonk, NY: IBM Corp). Normally distributed numerical data were presented as mean and SD, and skewed data were presented as median and interquartile range. Qualitative data were presented as number and percentage or ratio. Normally distributed numerical data were compared using the unpaired *t*-test. Skewed numerical data were compared using the Mann–Whitney test and categorical data were compared using Fisher’s exact test. *P*-value < 0.05 was considered statistically significant.

## Results

**Fig. 4.**
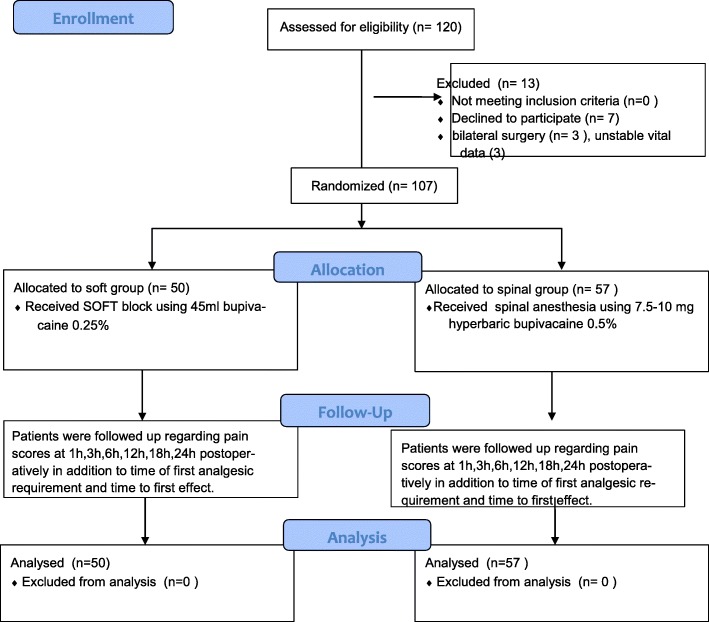
Study flow chart

A group of 120 patients were recruited for the study (Fig. 4), out of which 107 patients were randomised then analysed (50 patients received SOFT block and 57 received spinal anaesthesia). The research team decided to exclude patients from the study either because of clinical condition or violation of the protocol. Thirteen patients were excluded from the study because of patient’s withdrawal of consent (7), bilateral surgery (3), patients with unstable vital signs (3).

Demographic data, ASA status, comorbidities, and surgical data were comparable between the two study groups (Table 1).

**Table 1:** Demographic data

Demographic data	SOFT group (*n* = 50)	Spinal group (*n* = 57)	P-value
Sex			
Female	26(52%)	35(61%)	0.100
Age(years)	41.53 ± 6.84	43.1 ± 5.18	0.159
ASA			
I	29(58%)	42(74%)	0.559
II	21(42%)	15(26%)	
Surgical procedure duration(min)	207.62 ± 26.78	210.44 ± 32.98	0.608

All data were presented as percentage except age and procedural duration were presented as mean ± SD

The intention-to-treat analysis of the primary outcome revealed that the duration of analgesia in patients receiving SOFT block was (9.43 ± 2.7 h) compared with those receiving spinal anaesthesia which was (3.15 ± 1.83 min) (*p* < 0.001) (Table 2). Satisfaction scores were comparable between the study groups (*p* = 0.562) (Table 2). Non- significant difference between the study groups regarding the incidence of vomiting (*p* = 0.543) (Table 2). Only one patient of SOFT group had seizures. (Table 2). Non- significant difference between the study groups regarding the incidence of cardiovascular collapse (*p* = 0.591) and paraesthesia (*p* = 0.309) (Table 2).

**Table 2:** Comparison of the duration of analgesia, satisfaction scores and postoperative complications between the study groups

	SOFT group (*n* = 50)	Spinal group (*n* = 57)	*P*-value
Time to first analgesic requirement (hours)	9.43 ± 2.7	3.15 ± 1.83	< 0.001*
Time to first effect(min)	22.3 ± 2.07	8.03 ± 1.7	< 0.001*
Patient satisfaction scores	3.24 ± 0.64	3.3 ± 0.48	0.562
Postoperative complications			
Vomiting	5(10%)	7(12%)	0.543
Seizures	1(2%)	0(0.0%)	0.315
Cardiovascular collapse	7(14%)	9(16%)	0.591
Paraesthesia	3(6%)	1(2%)	0.309

All data were presented as percentage except the duration of block and satisfaction scores were presented as mean ± SD * *highly significant

The time to the first effect of SOFT was significantly longer in comparison to spinal anaesthesia (*p* < 0.001) (Table 2). Regarding visual analogue pain scores, there was no significant difference between the study groups at 1 h, 18 h, 24 h postoperative (Table 3). Visual analogue pain scores were remarkably lower in SOFT group compared with spinal group at 3 h (CI: 2.57 1-2.869),6 h (CI: 2.929–3.271) and 12 h (CI: 1.664–2.136) postoperatively (Table 3).

**Table 3:** Comparison of visual analogue scores between the study groups

Pain scores	SOFTgroup (*n* = 50)	Spinalgroup (*n* = 57)	*P* value	95% CI
1 h	1.12 ± 0.57	1.27 ± 0.45	0.131	0.046–0.346
3 h	1.35 ± 0.36	4.07 ± 0.41	< 0.001^a^	2.571–2.869
6 h	2.13 ± 0.52	5.23 ± 0.37	< 0.001^a^	2.929–3.271
12 h	5.19 ± 0.53	7.09 ± 0.68	< 0.001^a^	1.664–2.136
18 h	3.3 ± 0.5	3.37 ± 0.53	0.485	0.128–0.268
24 h	2.17 ± 0.48	2.24 ± 0.67	0.541	0.156–0.296

^a^ means highly significant

## Discussion

SOFT is a novel efficient nerve block that is found to accomplish excellent analgesia, its success rate is similar to the earlier discussed multiple nerve block techniques without significant side-effects [5]. However, it needs to be performed by well- trained skilled anaesthetist because of its complexity, so it is not a popular technique as spinal anaesthesia [3]. Good understanding of the regional anatomy and surgical technique is mandatory for the success of the nerve block [9].

This study is the first to compare the efficacy of SOFT with spinal anaesthesia in patients scheduled for elective surgery for fixation of open tibial fractures using Ilizarov external fixator.

The results of our study showed that SOFT block resulted in longer duration of analgesia, and better patient satisfaction scores and lower pain scores postoperatively with less incidence of adverse events as back pain and headache compared with spinal anaesthesia in patients undergoing lower limb surgery.

After literature review, it was found that few studies are consistent with our findings and confirm the analgesic efficacy and safety of this block. For example, a prospective study by Taha et al. was undertaken on 50 patients undergoing knee surgeries. The success rate was 90% of the patients resulting in complete anaesthesia. The median [IQR] patient pain scores on a numeric rating scale of 1–10 was 2, and the median [IQR] performance time was 5.5 min [5]. Moreover, A randomised controlled study by Felix and his colleagues using combined sciatic-femoral nerve block or low-dose spinal anaesthesia used for patients posted for knee arthroscopy. They reported that satisfaction scores in both groups were similar. Sciatic-femoral nerve blocks provided significantly lower pain scores particularly in the first 6 postoperative hours (*p* < 0.002) [10]. A prospective study by Sermin and his friends randomised 60 patients undergoing hallux valgus repair into spinal and popliteal groups. Complications as hypotension and bradycardia of 6and 3% were encountered in spinal anaesthesia group respectively. Regarding popliteal nerve block, there was minimal incidence of complications. Visual analogue scores were significantly lower in popliteal group compared with spinal group. Time of first rescue analgesic requirement was significantly longer in popliteal group [11]. These studies support the findings in our study.

A prospective study by Akcan et al. performed on 30 patients undergoing knee arthroplasty who were randomised into spinal or femoral-sciatic nerve block group. It concluded that spinal anaesthesia group showed significantly higher incidence of nausea and hypotension, lower satisfaction scores. Pain scores were significantly lower in ultrasound guided femoral- sciatic nerve block group [12].

Similarly, a randomised trial by Mc Namee and his collegues included 60 patients undergoing knee replacement surgery using femoral sciatic in addition to obturator block concluded that the duration of analgesia was significantly prolonged (433.06 min) with no incidence of any systemic of neurologic complications [13]. Our results are in agreement with the findings of Kim et al. who compared sciatic, femoral, and lateral cutaneous nerve blocks with combined spinal epidural anaesthesia in 84 patients scheduled for knee arthroscopy as regard intraoperative patient satisfaction and the duration of postoperative analgesia. Kim noticed that the satisfaction scores were higher in sciatic femoral and lateral cutaneous nerve block group [14].

Similar to our results, Spasiano et al. investigated the efficacy of combined sciatic and femoral blocks in 32 patients undergoing knee arthroscopy. They suggested that femoral sciatic nerve block is an interesting alternative to spinal anaesthesia because the incidence of postoperative complications was negligible [3].

Performance of multiple nerve blocks requires large doses of bupivacaine so we have to be alert about the risk of LA toxicity which causes convulsions at plasma level 4 μg/ml. For this reason, enantiomer LA with lower concentration has to be used [15] and we found that it theoretically provides adequate anaesthesia for this type of surgical procedure. Some studies aimed to reducing the number of nerves blocked to decrease the risk of toxicity but it may cause inadequate anaesthesia to perform the required surgical procedures [16].

## Limitations

Our study is subjected to a number of limitations including: Initially, we did not compare the success or failure rate of each block as all patients completed the study successfully (100% success rate) or the time of performance of each block. Moreover, inadequate earlier studies discussing the analgesic advantages of SOFT block as it is a novel technique so further randomised controlled studies need to be performed to prove these effects. In addition, few studies compare the efficacy of peripheral block techniques for the lower limb surgeries as an alternative to extradural or general anaesthesia, and most of them were case reports. Also, we didn’t use SOFT for anaesthetic management for surgical procedures of urgent context or prolonged duration. Additional studies are needed to calculate the optimal dose of LA used in combined nerve blocks depending on the nature and duration of the surgical procedure.

## Conclusions

Our results showed that SOFT block is a feasible technique of local anaesthesia for control of postoperative pain with unremarkable adverse events compared with spinal anaesthesia, in patients undergoing fixation of open tibial fractures using Ilizarov external fixator.

## Data Availability

The data sets used during the current study are available from the corresponding author on reasonable request.

## Abbreviations

ASA: American society of anesthesiologists
LA: Local anaesthetic
PASS: Power calculations and sample size software
SOFT: Sciatic obturator femoral technique
